# Draft genome sequences analyses of 25 NDM-producing *Escherichia coli* isolates from pediatric gut samples in Shenzhen, China

**DOI:** 10.1128/mra.00147-26

**Published:** 2026-04-20

**Authors:** Yunxing He, Tingjin Chen

**Affiliations:** 1 Guangdong Provincial Key Laboratory of Digestive Cancer Research, Digestive Diseases Center, Scientific Research Center, The Seventh Affiliated Hospital Sun Yat-sen University, Shenzhen, Guangdong, China; 2 Clinical Laboratory, Key Laboratory of Bacterial Resistance and Prevention, Medical Research Institute of Maternal and Child, Affiliated Shenzhen Women and Children’s Hospital (Longgang) of Shantou University Medical College (Longgang District Maternity & Child Healthcare Hospital of Shenzhen City), Shenzhen, Guangdong, China; Fluxus Inc., Sunnyvale, California, USA

**Keywords:** New Delhi metallo-β-lactamases, *Escherichia coli*, whole-genome sequencing

## Abstract

We report 25 carbapenem-resistant *Escherichia coli* strains isolated from pediatric gut samples in Shenzhen, China, including 24 isolates with *NDM*-5 and one isolate with *NDM*-1. Genome analysis aids understanding of resistance, virulence, and transmission, guiding infection control and therapies.

## ANNOUNCEMENT

Carbapenem-resistant Enterobacterales (CRE), particularly those producing New Delhi metallo-β-lactamase (*NDM*), pose a significant threat to pediatric health by limiting treatment options and facilitating community spread (1). In China, *NDM*-1 and *NDM*-5 are predominant variants in *Escherichia coli* (*E. coli*), often associated with multidrug resistance and gut colonization in children (2–4). Here, we announce the draft genome sequences of 25 *NDM*-producing *E. coli* isolates from pediatric fecal samples in Shenzhen, China, to support genomic surveillance and comparative analyses.

In 2024, 1,189 non-duplicate fecal specimens from children were screened at Longgang District Maternity & Child Healthcare Hospital (22.7303°N, 114.2301°E) (4), Shenzhen, China. Approximately 1 g of each fecal specimen was suspended in 9 mL of sterile phosphate-buffered saline, vortexed vigorously for 1 min, and 100 μL of the suspension was directly plated onto MacConkey agar supplemented with 1.5 μg/mL imipenem, and incubated at 37°C with 5% CO₂ for 48 h. Strains were identified using matrix-assisted laser desorption/ionization time-of-flight mass spectrometry (Bruker Corporation, Billerica, MA, USA) and screened for *NDM* by PCR using primers *NDM*-F (5′-GGTTTGGCGATCTGGTTTTC-3′) and *NDM*-R (5′-CGGAATGGCTCATCACGATC-3′) (5). This yielded 25 *NDM*-positive isolates, which were stored at −80°C in Luria-Bertani broth with 30% glycerol. Ethical approval was obtained from the Ethics Committee of Shantou University Medical College (KYXM-2023-052); no patient consent was required as the study used anonymized isolates. The isolates were revived from the −80°C glycerol stocks by streaking onto blood agar plates and incubated overnight at 37°C. Genomic DNA was then extracted using the SteadyPure Bacterial Genomic DNA Extraction Kit (Accurate Biology, AG21007, China) according to the manufacturer’s instructions. The quality and quantity of the extracted genomic DNA were evaluated using a Qubit Fluorometer for concentration measurement and agarose gel (0.5%) electrophoresis for sample integrity assessment according to the manufacturer’s protocols (6, 7). Sequencing libraries were prepared using the AccuNext CUT&Tag DNA Library Kit (Accurate Biology, AG12556, China) according to the manufacturer’s protocol with no modifications and sequenced on Illumina NovaSeq 6000 (2 × 150 bp paired-end reads), generating a total of approximately 250 million reads across all 25 genomes (average 10 million reads per isolate), corresponding to ~300 × coverage. Raw reads were quality-filtered with fastp v0.20.1 (8) with default parameters, yielding clean reads. The genomes were assembled using SKESA v. 2.2 with default parameters. Assemblies were annotated with the NCBI Prokaryotic Genome Annotation Pipeline (PGAP v6.10) using default parameters.

Assembly statistics for the 25 *NDM*-producing *E. coli* draft genomes are summarized in Table 1. Multilocus sequence typing (MLST) via EnteroBase (9) revealed diverse lineages, dominated by ST48 (ST10 complex; *n* = 6), followed by ST648 (*n* = 2), ST101 (*n* = 2), and ST155 (*n* = 2); one novel ST (ST18136) was identified. Resistance gene profiling with the Comprehensive Antibiotic Resistance Database (CARD) (10) confirmed *NDM*-5 in 24 isolates (96%) and *NDM*-1 in one isolate (4%). These genomes provide insights into the genomic diversity of pediatric *NDM*-producing *E. coli* in China, highlighting multidrug resistance and colonization potential.

**TABLE 1:** Assembly statistics for draft genomes of 25 *NDM*-producing *E. coli* isolates^*a*^

Name	Coverage (x)	N50 (kb)	Genome size (bp)	Contig number	Gene number	RNA number	GC content (%)	Clean reads	MLST (clonal complex)	*NDM* typing	Enterobase	NCBI
CNSZ24HYX	527	81.4	4,712,575	188	4700	90	51.0	13,499,722	10 (ST10 Cplx)	*NDM*-5	ESC_VB4161AA	SAMN54299096
CNSZ1HYX	312	322.1	5,332,420	158	5269	89	50.5	8,894,020	648 (ST10 Cplx)	*NDM*-5	ESC_WB1423AA	SAMN54242561
CNSZ41HYX	325	118.8	5,301,702	155	5260	98	50.5	9,089,294	69 (ST69 Cplx)	*NDM*-5	ESC_WB1424AA	SAMN54299080
CNSZ48HYX	369	188.8	4,788,203	149	4753	96	51.0	9,289,898	48 (ST10 Cplx)	*NDM*-5	ESC_VB4158AA	SAMN54299097
CNSZ74HYX	268	212.6	5,460,465	156	5398	95	50.5	7,607,920	648 (ST10 Cplx)	*NDM*-5	ESC_WB1421AA	SAMN54299081
CNSZ104HYX	314	94.9	4,784,046	137	4742	94	51.0	7,672,082	48 (ST10 Cplx)	*NDM*-5	ESC_WB1422AA	SAMN54299082
CNSZ144HYX	324	77.4	4,951,541	183	5003	95	50.5	8,383,550	48 (ST10 Cplx)	*NDM*-5	ESC_WB1428AA	SAMN54299083
CNSZ145HYX	349	77.4	4,947,067	182	5001	96	50.5	8,794,114	48 (ST10 Cplx)	*NDM*-5	ESC_WB1432AA	SAMN54299084
CNSZ152HYX	301	76.5	4,910,204	178	4956	95	51.0	7,521,420	48 (ST10 Cplx)	*NDM*-5	ESC_WB1426AA	SAMN54299085
CNSZ171HYX	334	155.5	5,000,867	134	4963	96	50.5	8,515,266	641 (ST86 Cplx)	*NDM*-5	ESC_WB1430AA	SAMN54299086
CNSZ209HYX	327	63.7	5,060,004	282	5226	102	50.5	8,716,026	6215 (-)	*NDM*-5	ESC_WB1429AA	SAMN54299087
CNSZ228HYX	312	143.7	5,115,604	143	5210	97	50.5	8,178,912	101 (ST101 Cplx)	*NDM*-5	ESC_WB1427AA	SAMN54299088
CNSZ231HYX	360	108.9	5,071,804	156	5023	87	50.5	9,720,582	58 (ST155 Cplx)	*NDM*-5	ESC_WB1433AA	SAMN54299089
CNSZ240HYX	314	121.1	5,301,030	275	5502	109	50.5	8,737,388	40 (ST40 Cplx)	*NDM*-5	ESC_WB1431AA	SAMN54242562
CNSZ249HYX	295	153.3	4,987,287	156	4942	96	50.5	7,444,484	515 (-)	*NDM*-5	ESC_WB1465AA	SAMN54299090
CNSZ376HYX	343	75.5	5,061,027	203	5088	94	50.5	8,770,988	18,136 (ST168 Cplx)	*NDM*-5	ESC_UB2001AA	SAMN54299095
CNSZ578HYX	334	79.8	4,940,240	169	4902	91	50.5	8,478,376	48 (ST10 Cplx)	*NDM*-1	ESC_WB1470AA	SAMN54299091
CNSZ589HYX	365	142.8	5,353,230	176	5341	91	50.5	10,091,084	410 (ST23 Cplx)	*NDM*-5	ESC_VB4160AA	SAMN54299098
CNSZ650HYX	358	137.2	5,121,568	115	5084	92	50.5	9,320,354	155 (ST155 Cplx)	*NDM*-5	ESC_WB1471AA	SAMN54299092
CNSZ794HYX	407	183.2	4,892,529	91	4874	94	50.5	10,343,272	101 (ST101 Cplx)	*NDM*-5	ESC_WB1472AA	SAMN54299093
CNSZ841HYX	337	162.8	5,088,795	181	5177	97	50.5	8,652,032	154 (-)	*NDM*-5	ESC_WB1674AA	SAMN54299094
CNSZ1022HYX	396	92.5	5,113,247	203	5197	95	50.5	10,341,350	216 (-)	*NDM*-5	ESC_WB1675AA	SAMN54299078
CNSZ1038HYX	365	161.4	5,035,444	150	5006	97	50.5	9,367,510	154 (-)	*NDM*-5	ESC_VB4159AA	SAMN54299099
CNSZ1053HYX	318	180.5	5,147,891	112	5076	85	50.5	8,164,966	457 (-)	*NDM*-5	ESC_WB1673AA	SAMN54299079
CNSZ1113HYX	286	150.5	5,231,222	135	5190	104	50.5	7,624,748	38 (ST38 Cplx)	*NDM*-5	ESC_WB1672AA	SAMN54249838

^*a*^ The genome accessions listed in the Enterobase database are assemblies generated from raw sequencing data, while the corresponding NCBI accessions refer to the unassembled raw reads.

## Data Availability

Raw reads are available in the NCBI Sequence Read Archive (SRA, https://www.ncbi.nlm.nih.gov/sra/?term=) under the following accession numbers: SAMN54299096, SAMN54242561, SAMN54299080, SAMN54299097, SAMN54299081, SAMN54299082, SAMN54299083, SAMN54299084, SAMN54299085, SAMN54299086, SAMN54299087, SAMN54299088, SAMN54299089, SAMN54242562, SAMN54299090, SAMN54299095, SAMN54299091, SAMN54299098, SAMN54299092, SAMN54299093, SAMN54299094, SAMN54299078, SAMN54299099, SAMN54299079, and SAMN54249838. The assembled and annotated genome sequences generated in this project have been uploaded to the EnteroBase database (https://enterobase.warwick.ac.uk/species/index/ecoli).

## References

[1] Wu W, Feng Y, Tang G, Qiao F, McNally A, Zong Z. 2019. NDM metallo-β-lactamases and their bacterial producers in health care settings. Clin Microbiol Rev 32:e00115–00118. doi:10.1128/CMR.00115-18 30700432 PMC6431124

[2] Han R, Shi Q, Wu S, Yin D, Peng M, Dong D, Zheng Y, Guo Y, Zhang R, Hu F, China Antimicrobial Surveillance Network (CHINET) Study Group. 2020. Dissemination of carbapenemases (KPC, NDM, OXA-48, IMP, and VIM) among carbapenem-resistant Enterobacteriaceae isolated from adult and children patients in China. Front Cell Infect Microbiol 10:314. doi:10.3389/fcimb.2020.00314 32719751 PMC7347961

[3] Yang H, Xiong Z, Cao K, He Y, Song S, Lan F, Yang K, Liu X, Duan C, Zhou Z. 2025. Risk factors and molecular epidemiology of colonizing carbapenem-resistant Enterobacterales in pediatric inpatient in Shenzhen, China. J Infect Public Health 18:102614. doi:10.1016/j.jiph.2024.102614 39642772

[4] He Y, Yang H, Liao C, Ou X, Yang K, Ding T, Zhou X, Liu X, Liu S, Zhou Z, Chen T. 2026. Phenotypic and genomic characterization of NDM-producing Escherichia coli colonizing the pediatric gut in Shenzhen, China. Microbiol Spectr 14:e0332925. doi:10.1128/spectrum.03329-25 41685987 PMC13055255

[5] Poirel L, Walsh TR, Cuvillier V, Nordmann P. 2011. Multiplex PCR for detection of acquired carbapenemase genes. Diagn Microbiol Infect Dis 70:119–123. doi:10.1016/j.diagmicrobio.2010.12.002 21398074

[6] Thermo Fisher Scientific. 2023. Qubit dsDNA HS assay kit user guide. Carlsbad, CA: Thermo Fisher Scientific.

[7] Green MR, Sambrook J. 2012. Molecular cloning: a laboratory manual. 4th ed. Cold Spring Harbor, NY: Cold Spring Harbor Laboratory Press.

[8] Chen S, Zhou Y, Chen Y, Gu J. 2018. Fastp: an ultra-fast all-in-one FASTQ preprocessor. Bioinformatics 34:i884–i890. doi:10.1093/bioinformatics/bty560 30423086 PMC6129281

[9] Jolley KA, Bray JE, Maiden MCJ. 2018. Open-access bacterial population genomics: BIGSdb software, the PubMLST.org website and their applications. Wellcome Open Res 3:124. doi:10.12688/wellcomeopenres.14826.1 30345391 PMC6192448

[10] Alcock BP, Huynh W, Chalil R, Smith KW, Raphenya AR, Wlodarski MA, Edalatmand A, Petkau A, Syed SA, Tsang KK, et al.. 2023. CARD 2023: expanded curation, support for machine learning, and resistome prediction at the comprehensive antibiotic resistance database. Nucleic Acids Res 51:D690–D699. doi:10.1093/nar/gkac920 36263822 PMC9825576

